# A novel thrombocytopenia‐4‐causing CYCS gene variant decreases caspase activity: Three‐generation study

**DOI:** 10.1111/bjh.19694

**Published:** 2024-08-27

**Authors:** Jiří Štika, Michaela Pešová, Kateřina Staňo Kozubík, Magdalena Skalníková, Lenka Dostálová, Tomáš Loja, Lenka Radová, Veronika Palušová, Kamila Réblová, Zuzana Vrzalová, Ivona Blaháková, Jakub Trizuljak, Stjepan Uldrijan, Jan Blatný, Michal Šmída, Šárka Pospíšilová, Michael Doubek

**Affiliations:** ^1^ Center of Molecular Medicine CEITEC – Central European Institute of Technology, Masaryk University Brno Czechia; ^2^ Institute of Medical Genetics and Genomics Faculty of Medicine, Masaryk University and University Hospital Brno Brno Czechia; ^3^ Department of Internal Medicine – Hematology and Oncology Faculty of Medicine, Masaryk University and University Hospital Brno Brno Czechia; ^4^ Department of Biology Faculty of Medicine, Masaryk University Brno Czechia; ^5^ International Clinical Research Center St. Anne's University Hospital Brno Czechia; ^6^ Department of Pediatric Hematology and Biochemistry University Hospital Brno Brno Czechia

**Keywords:** caspase, CRISPR/Cas9, CYCS, cytochrome c, mitochondria, thrombocytopenia

## Abstract

The *CYCS* gene is highly evolutionarily conserved, with only a few pathogenic variants that cause thrombocytopenia‐4 (THC4). Here, we report a novel *CYCS* variant NM_018947.6: c.59C>T [NP_061820.1:p.(Thr20Ile)] segregating with thrombocytopenia in three generations of a Czech family. The phenotype of the patients corresponds to THC4 with platelets of normal size and morphology and dominant inheritance. Intriguingly, a gradual decline in platelet counts was observed across generations. CRISPR/Cas9‐mediated gene editing was used to introduce the new *CYCS* gene variant into a megakaryoblast cell line (MEG‐01). Subsequently, the adhesion, shape, size, ploidy, viability, mitochondrial respiration, cytochrome c protein (CYCS) expression, cell surface antigen expression and caspase activity were analysed in cells carrying the studied variant. Interestingly, the variant decreases the expression of CYCS while increasing mitochondrial respiration and the expression of CD9 cell surface antigen. Surprisingly, the variant abates caspase activation, contrasting with previously known effects of other *CYCS* variants. Some reports indicate that caspases may be involved in thrombopoiesis; thus, the observed dysregulation of caspase activity might contribute to thrombocytopenia. The findings significantly enhance our understanding of the molecular mechanisms underlying inherited thrombocytopenia and may have implications for diagnosis, prognosis and future targeted therapies.

## Graphical abstract

Thrombocytopenia 4 is a sporadic form of inherited thrombocytopenia caused by variants in the *CYCS* gene (cytochrome c). The new *CYCS* variant NM_018947.6: c.59C>T [NP_061820.1:p.(Thr20Ile)] segregates with thrombocytopenia in three generations of the Czech family. CRISPR/Cas9‐mediated gene editing was used to introduce the *CYCS* gene variant into a megakaryoblast cell line (MEG‐01). Subsequently, the adhesion, shape, size, ploidy, viability, mitochondrial respiration, cytochrome c protein (CYCS) expression, cell surface antigen expression and caspase activity were analysed. The variant interferes with protein expression, increases mitochondrial activity and reduces basal caspase activity, a reduction that is distinct among thrombocytopenia‐causing *CYCS* gene variants.

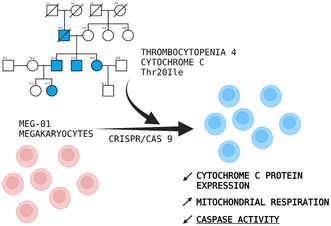

## INTRODUCTION

Mutations in over 40 genes have been described as causative of inherited thrombocytopenia (IT), including certain variants that increase the risk of malignancy. Molecular characterization is, therefore, crucial for the diagnosis, prognosis and possible prevention or treatment of IT. Thrombocytopenia‐4 (THC4; OMIM 612004) is a sporadic form of IT caused by variants in the *CYCS* gene. THC4 manifests as mild to moderate autosomal dominant thrombocytopenia with normal platelet size, morphology and function. Only seven *CYCS* variants have been described so far: p.(His27Tyr), p.(Gly42Ser), p.(Tyr49His), p.(Ala52Val), p.(Arg92Gly), p. (Leu99Val) and p.(Lys101del).^1^, ^2^, ^3^, ^4^, ^5^, ^6^, ^7^ Of these, three novel variants have been described only very recently as part of the largest series of THC4 cases reported to date.^8^

Cytochrome c protein (CYCS), the product of the *CYCS* gene, activates caspases.^9^ Localized caspase activation and the release of CYCS from mitochondria are crucial for proplatelet formation in human bone marrow CD34^+^‐derived megakaryocytes.^10^ Activation of caspases also leads to proplatelet production in the MEG‐01 megakaryoblastic cell line.^11^ These findings pointed to a critical role of CYCS and caspases in human thrombopoiesis.

Here, we report a functional analysis of the novel CYCS variant NP_061820.1:p.(Thr20Ile) (NM_018947.6:c.59C>T), which segregates with thrombocytopenia in three generations of a Czech family. The variant is characterized as likely pathogenic according to the guidelines and criteria set forth by the American College of Medical Genetics and Genomics and the Association for Molecular Pathology (ACMG/AMP)^12^ It disrupts protein structure by in silico analysis. Because thrombocytopenia could result from impaired control of apoptosis and differentiation of megakaryocytes, events potentially involved in these processes, such as CYCS expression, adhesion, shape, size, ploidy, viability, mitochondrial respiration, caspase activation and expression of cell surface antigens, were studied in MEG‐01 cells modified to carry CYCS variant p.(Thr20Ile). Using the MEG‐01 model allowed us to avoid invasive procedures for patients, such as bone marrow aspiration. The variant decreases CYCS expression, increases the levels of respiration and expression of CD9 cell surface antigen and decreases caspase activation, which may lead to impaired thrombopoiesis.

## MATERIALS AND METHODS

### Study approval

Patients or their legal guardians signed written informed consent in accordance with the Declaration of Helsinki and protocol approved by institutional ethics committees (Masaryk University and the University Hospital Brno, Czechia).

### Variant screening

Four thrombocytopenic (II‐1, III‐3, III‐4 and IV‐2) and three thrombocytopenia‐free (II‐2, III‐2 and IV‐1) family members were selected for whole exome sequencing (WES) analysis to uncover the thrombocytopenia‐causing variants.

Genomic DNA was isolated from peripheral blood using the MagCore® Genomic DNA Whole Blood Kit (RBC Bioscience). Whole exome libraries were prepared by the KAPA Hyper Prep Kit, SeqCap EZ Human Exome Probes v3, and HyperCap Bead Kit (Roche) according to the SeqCap EZ HyperCapWorkflow v2.1. Paired‐end 2 × 150 bp sequencing was performed on the Illumina NextSeq 500 Sequencer (Illumina, Inc.). Sequencing data met set internal QC standards: 90% of reads were mapped to regions of interest, with a coverage >30×. The FastQC tool was applied to the quality checks of sequenced samples. The raw sequencing reads were aligned to the GRCh38 reference genome using BWA‐mem (version 0.7.15). PCR duplicates were identified using the Mark‐Duplicates tool from Picard. Germline single nucleotide variants (SNVs) and indels were detected by local reassembly of haplotypes in GATK HaplotypeCaller v3.7. Annotation of the variants/indels obtained was performed with Annovar. Only variants with a total coverage of at least 15× were included. Processed SNVs/indels were compared in affected versus healthy family members. Additionally, all affected family members (II‐1, III‐3, III‐4 and IV‐2) were tested for the presence of variants in 5′ UTR of the *ANKRD26* gene by Sanger sequencing with negative results.

The *CYCS* gene variant identified by WES was confirmed by Sanger sequencing on the 3500 Genetic Analyser (Applied Biosystems) using F‐primer: TGCTTGGGCTTTAACGTTCC, R‐primer: CATCGGTTATTTCACACTCCTGAT and a BigDye Terminator v3.1 Cycle Sequencing Kit according to the manufacturer's protocol.

The variant was classified using ACMG/AMP guidelines. Variant effect prediction was based on prediction tools: Align GVGD (http://agvgd.hci.utah.edu/agvgd_input.php), Mutation taster (https://www.mutationtaster.org/) and SIFT (https://sift.bii.a‐star.edu.sg/index.html).

Finally, in silico analysis of the crystal structure of human CYCS with high resolution 1.35 Å (3ZOO.pdb)^13^ was performed using the VMD programme.^14^ The protein contains 5 α‐helices and 3 conserved Ω‐loops.^15^

### Cell culture and transfection

A megakaryoblastic human cell line, MEG‐01, was chosen as a model because it is the closest to the naturally occurring cell lineage in the human body that gives rise to platelets. The cell line was purchased from the American Type Culture Collection (ATCC). The cells were grown in RPMI‐1640 (Merck) supplemented with 10% fetal bovine serum (Merck) and 1% penicillin–streptomycin (Merck) at 37°C, 5% CO_2_ and 95% humidity.

To introduce *CYCS* (c.59C>T) substitution into the MEG‐01 cell line, the ribonucleoprotein (RNP) complexes and homology‐directed repair (HDR) template were electroporated into the cells using the NeonTM electroporation system (Thermo Fisher Scientific). Single guide RNA (sgCYCS: TGCCTCCCTTTTCAACGGTG; Synthego) was mixed with Cas9 2NLS nuclease protein (Synthego) and incubated at room temperature for 15 min to form RNP complexes. HDR template (Alt‐R HDR donor oligo from IDT) containing the variant of interest and protospacer adjacent motif (PAM) (ATGTTGAGAAAGGCAAGAAGATTTTTATTATGAAGTGTTCCCAGTGtCACAtCGTTGAAAAGGGcGGCAAGCACAAGACTGGGCCAAATCTCCATGGTCTCTT) was added to the RNP complex, and the solution was mixed with cells. Cells were electroporated using the following conditions: 1400 V, 20 ms, one pulse. The GFP‐expressing plasmid (pCMV‐GFP, a gift from Connie Cepko; Addgene plasmid #11153) was added to the solution as the marker of successful transfection.

The next day, after electroporation, GFP‐positive single cells were sorted into 96‐well plates using BD FACSAria Fusion cell sorter (BD Biosciences). After expanding the cells, genomic DNA was extracted, the site containing the variant of interest was amplified by PCR using specific primers (F: AGGTTCCCTCTTTGCTTGGG, R: GCATCGGTTATTTCACACTCCTG), and the variant presence was confirmed by Sanger sequencing. Clones designated SC1 and SC3 were selected for subsequent analyses. The adhesion, shape, size and viability (trypan‐blue staining) of cells were regularly assessed during cultivation by light microscope. Cell populations with viability over 90% were used in the assays.

### Western blotting

Peripheral blood samples from the affected patient (IV‐2) and the healthy controls (without thrombocytopenia and wild type/wt/*CYCS*) were collected in sodium citrate tubes, and platelet isolation was performed using the density gradient centrifugation and washing method.^16^ According to the flow cytometric analysis, platelet samples contained less than 1% leucocytes (CD45^+^) and more than 99% platelets (CD9^+^, CD41^+^ and CD61^+^).

The cytosolic protein fraction of MEG‐01 cells and platelets was prepared by NE‐PER nuclear and cytoplasmic extraction reagents kit (Thermo Fisher Scientific). Equal amounts of cytoplasmic lysates were mixed with Laemmli sample buffer (Bio‐Rad), boiled for 5 min and subjected to sodium dodecyl sulphate‐polyacrylamide gel electrophoresis. Proteins were subsequently transferred to the nitrocellulose membrane. The following antibodies were used for detection: rabbit anti‐cytochrome c (abcam; #ab133504), rabbit anti‐β‐Actin (Cell Signalling; #4970) and horseradish peroxidase‐linked anti‐rabbit IgG (Cell Signalling; #7074). The intensity of the bands was quantified by optical density using Uvitec Alliance Instrument and Uvitec Alliance software.

### Metabolic assays

Metabolic parameters were assessed in the Seahorse XFp Analyser using the XFp Cell Mito Stress Test Kit (Agilent), according to the manufacturer's instructions. One day before the measurement, MEG‐01 cells were seeded into XFp Poly‐d‐lysine Coated Cell Culture Microplates at a density of 2.5 × 10^4^ per well. One hour before the measurement, the culture medium was replaced with XF RPMI Medium pH 7.4 supplemented with 10 mM glucose, 2 mM glutamine and 1 mM pyruvate, and the microplates were transferred to a 37°C non‐CO_2_ incubator. Metabolic drugs were sequentially loaded into each experimental well of the microplates in the following order: 1.5 μM oligomycin, 1 μM carbonyl cyanide‐4‐(trifluoromethoxy)phenylhydrazone (FCCP) and 0.5 μM rotenone/antimycin A. The test was performed in three sequential measurements for basal oxygen consumption, followed by three measurements after administering each metabolic drug. Data were normalized to the cell number, measured by Attune Acoustic Focusing Cytometer (Thermo Fisher Scientific), and analysis was performed using the Seahorse Wave software.

### Caspase activity—Cell‐free assay

The Caspase‐Glo® 3/7—luminescent assay measuring caspase 3 and caspase 7 activities was prepared according to the manufacturer's guidelines. Cytoplasmic lysates were prepared from MEG‐01 cells in the same way as described in the western blotting section. All lysates were diluted to a concentration of 500 μg/mL, and 100 μL of reaction buffer was added to 100 μL of diluted lysate. Luminescence was measured at 10‐min intervals for 4 h using an automated fluorometer SPARK 10M (Tecan).

### Flow cytometry

#### Cell cycle analysis

For cell cycle analysis, MEG‐01 cells were harvested, washed with phosphate‐buffered saline (PBS) and fixed with 70% ethanol for 30 min at 4°C. Cells were washed twice with PBS and stained in Vindelov's reagent with 40 μg/mL RNAse A and 50 μg/mL propidium iodide for 30 min in the dark at 37°C. Cells were analysed on the BD FACSVerse—BD FACSuite 1.0.6 (BD Biosciences).

#### Cell surface antigen expression

Expression of cell surface antigens was analysed in MEG‐01 cells and platelets of patient IV‐2 with corresponding antibodies: CD9 (BD Biosciences; #561329), CD41 (Invitrogen; #12‐0419‐42) and CD61 (Sony Biotechnology; #2282020). For platelets, immunophenotyping was performed also using CD42a (Beckman Coulter; #IM1757U), CD42b (Invitrogen; #MHCD42B01) and CD49b (Invitrogen; #12–5971‐82) antibodies. The contamination of platelet samples with leucocytes before western blotting was checked by detecting the leucocyte marker CD45 (Sony Biotechnology; #2120070).

Unlabelled samples were used as negative controls. All samples were analysed on the BD FACSVerse—BD FACSuite 1.0.6.

#### Caspase activity

In addition to the cell‐free assay, the activation of caspases 3 and 7 in MEG‐01 cells was also studied using the CellEvent™ Caspase‐3/7 Green Flow Cytometry Assay Kit (Invitrogen) that measures the activity of caspases by cleavage of the fluorogenic substrate.

The procedure was performed according to the manufacturer's guidelines with minor modifications. Briefly, 1 μl of CellEvent™ Caspase‐3/7 Green Detection Reagent was added to cells in 0.5 mL of PBS with 2% bovine serum albumin and incubated for 30 min at 37°C, protected from light. During the final 5 min of staining, 0.5 μL of 1 mM SYTOX™ AADvanced™ dead cell stain solution in DMSO was added to distinguish dead cells. The samples were analysed using BD FACSVerse—BD FACSuite 1.0.6 (BD Biosciences). Caspase activity was evaluated in the population of living cells.

### Statistical analysis

All statistical analyses were performed in R.^17^ Unless otherwise stated, the ANOVA chi‐square test was performed to check the overall effect of the variables on the dependent variable in the logistic model. *p*‐Value of 0.05 was considered statistically significant, and *p*‐value of 0.10 was considered a trend.

## RESULTS

### Clinical features

**FIGURE 1:**
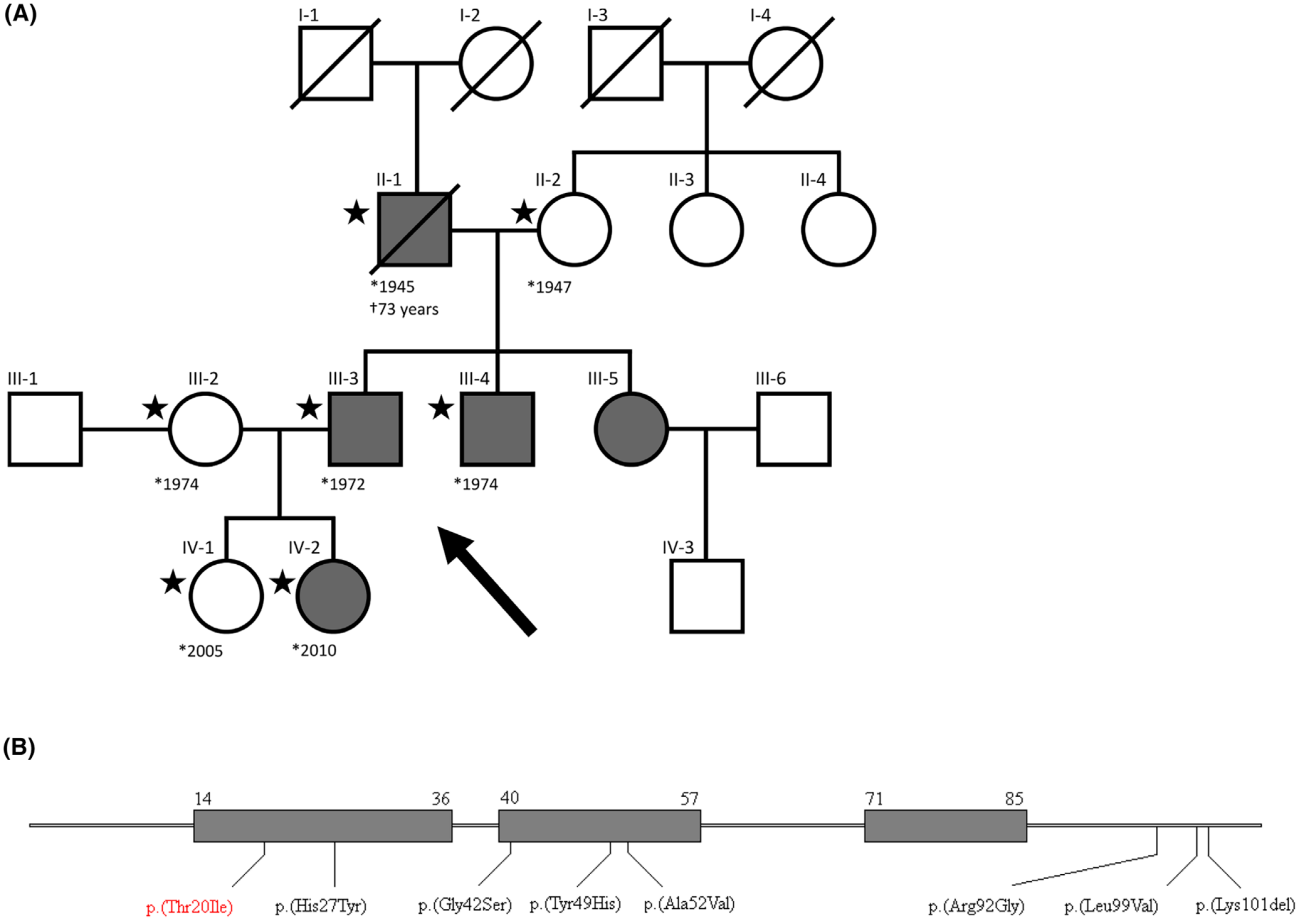
(A) The family pedigree shows autosomal dominant segregation of p.(Thr20Ile) variant with thrombocytopenia (dark filling). Asterisks indicate the samples used for whole exome sequencing. (B) Linear representation of CYCS (NP_061820.1) with already described THC4‐causing variants, including the variant described in this study (red). Three grey rectangles stand for Ω‐loops, numbers indicate the positions of the first and last amino acids of the loops.

The proband (III‐3), a 51‐year‐old male, has been followed for moderate thrombocytopenia since childhood. His condition was initially misdiagnosed as idiopathic thrombocytopenic purpura. He suffered from larger haematomas after injuries. There was no pathology in white blood count, and no other bleeding episodes. Thus, the proband was without medication. When his daughter was also found to have thrombocytopenia, the proband's diagnosis was reclassified as IT and the proband and his family were examined by a clinical geneticist at our institute. Figure 1A provides a pedigree of the reported family.

Patient IV‐2 (daughter of III‐3), a 13‐year‐old female, suffers from petechia and mild haematomas. Despite severe thrombocytopenia, bleeding episodes have rarely been reported. She was followed from 2012 to 2021 without requiring any medication. In 2021, she developed health problems requiring treatment with infliximab, azathioprine and ursodeoxycholic acid.

Blood tests of other family members revealed that two (II‐1 and III‐4) had thrombocytopenia, which they were not aware of. The other three (II‐2, III‐2 and IV‐1) showed no signs of thrombocytopenia. Thrombocytopenia was moderate in the first generation (II‐1), moderate/severe in the second (III‐3, III‐4) and severe in the third (IV‐2). Platelets were of normal morphology (Figure S1) and size. Table 1 summarizes platelet test results and clinical features.

**TABLE 1:** Platelet test results and clinical features of family members.

	Platelet count	MPV (fL)	ISTH/SSC bleeding score	Additional diagnoses
II‐1	**69 × 10** ^**9**^ **/L**	8.84	ND	UNK
II‐2	211 × 10^9^/L	10.50	ND	UNK
III‐2	277 × 10^9^/L	12.0	ND	UNK
III‐3	**53 × 10** ^**9**^ **/L**	10.40	1 cutaneous	Chronic autoimmune pancreatitis
III‐4	**55 × 10** ^**9**^ **/L**	10.70	ND	UNK
IV‐1	305 × 10^9^/L	10.10	ND	UNK
IV‐2	**20 × 10** ^**9**^ **/L**	NA^a^	2 cutaneous	Autoimmune sclerosing cholangitis, chronic cholestatic hepatopathy with mild fibrosis, ulcerative colitis and lactase deficiency

*Note*: Platelet count of thrombocytopenic patients is marked in bold font.

Abbreviations: ISTH, the International Society on Thrombosis and Haemostasis; MPV, mean platelet volume; ND, not done; SSC, Scientific and Standardization Committee; UNK, unknown.

^a^ NA: not analysed due to low platelet count.

### Identification of the variant p.(Thr20Ile)

We performed WES analysis to identify the genetic cause of thrombocytopenia in this family (Figure 1A). From resulting variants in genes associated with IT and haematopoiesis (Table S1), present in all thrombocytopenic family members and not present in healthy family members, only heterozygous variant within *CYCS* exon 2 [NM_018947.6:c.59C>T, NP_061820.1:p.(Thr20Ile)] was identified as potentially causing IT. The variant was classified as likely pathogenic according to the ACMG/AMP criteria (PM2, PP1–4). The c.59C>T variant was absent in the analysed thrombocytopenia‐free family members. This variant is not present in GnomAD (v4.1.0) and Human gene mutation database (HGMD; Professional v.2024.2). Results were confirmed by Sanger sequencing.

Furthermore, the effect of the variant was evaluated in silico. According to Align GVGD, the variant most likely interferes with the protein function (class C65). MutationTaster predicted the variant to be disease‐causing (probability 99.998%), and according to SIFT, the variant affects protein function (score 0.00).

To inspect the possible effect of the variant in more detail, we have calculated that threonine at position 20 occurs in 94% of the 113 different eukaryote *CYCS* sequences published by Banci et al.,^18^ with no isoleucine naturally occurring at this position. The phylogenetic conservation of the amino acid at position 20 further strongly supports the predicted deleterious effect of the variant.

**FIGURE 2:**
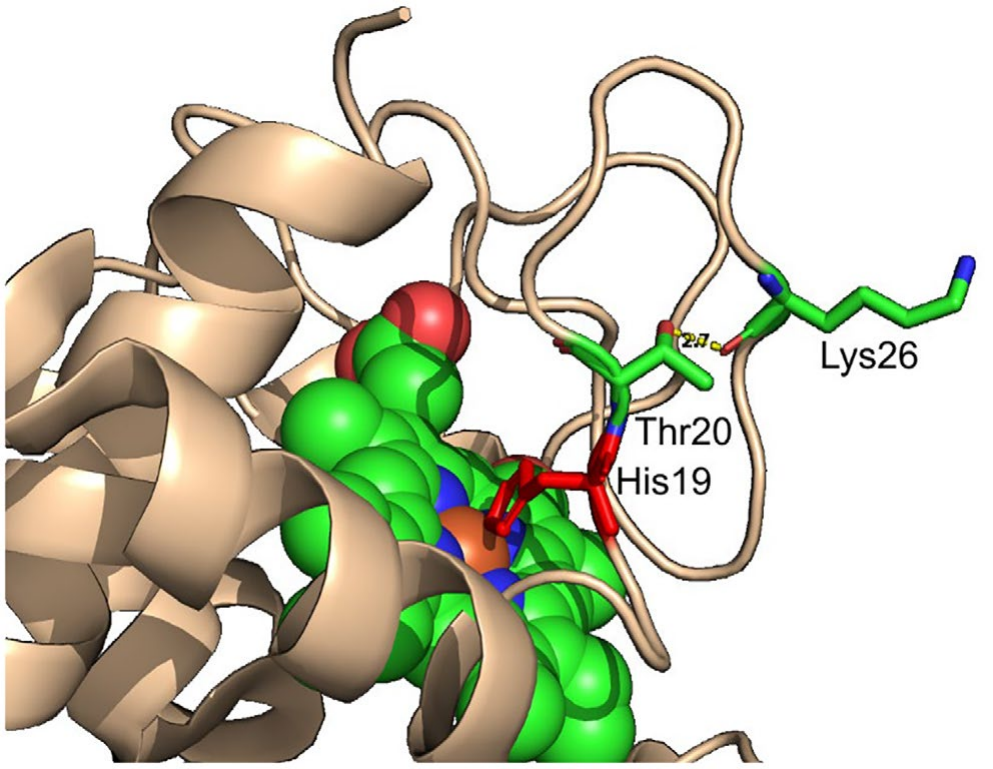
Human crystal structure of N‐terminal domain of CYCS (PDB code: 3ZOO). Thr20 is highlighted in liquorice representation and creates an H‐bond to Lys26. Adjacent His19 (in red) binding haem iron is also shown.

Next, CYCS in silico crystal structure analysis (pdb code: 3ZOO) revealed that the variant p.(Thr20Ile) could cause the loss of native H‐bond contact between wt Thr20(OH) and Lys26(O). In addition, the binding of adjacent His19 and Fe^2+^ in the active site of the protein may also be disrupted (Figure 2).

Despite the immunity issues in III‐3 and IV‐2 family members, no pathogenic or likely pathogenic genetic variants in immunity‐related genes (Table S2) have been found in the analysed family members.

### The variant p.(Thr20Ile) reduces CYCS expression

**FIGURE 3:**
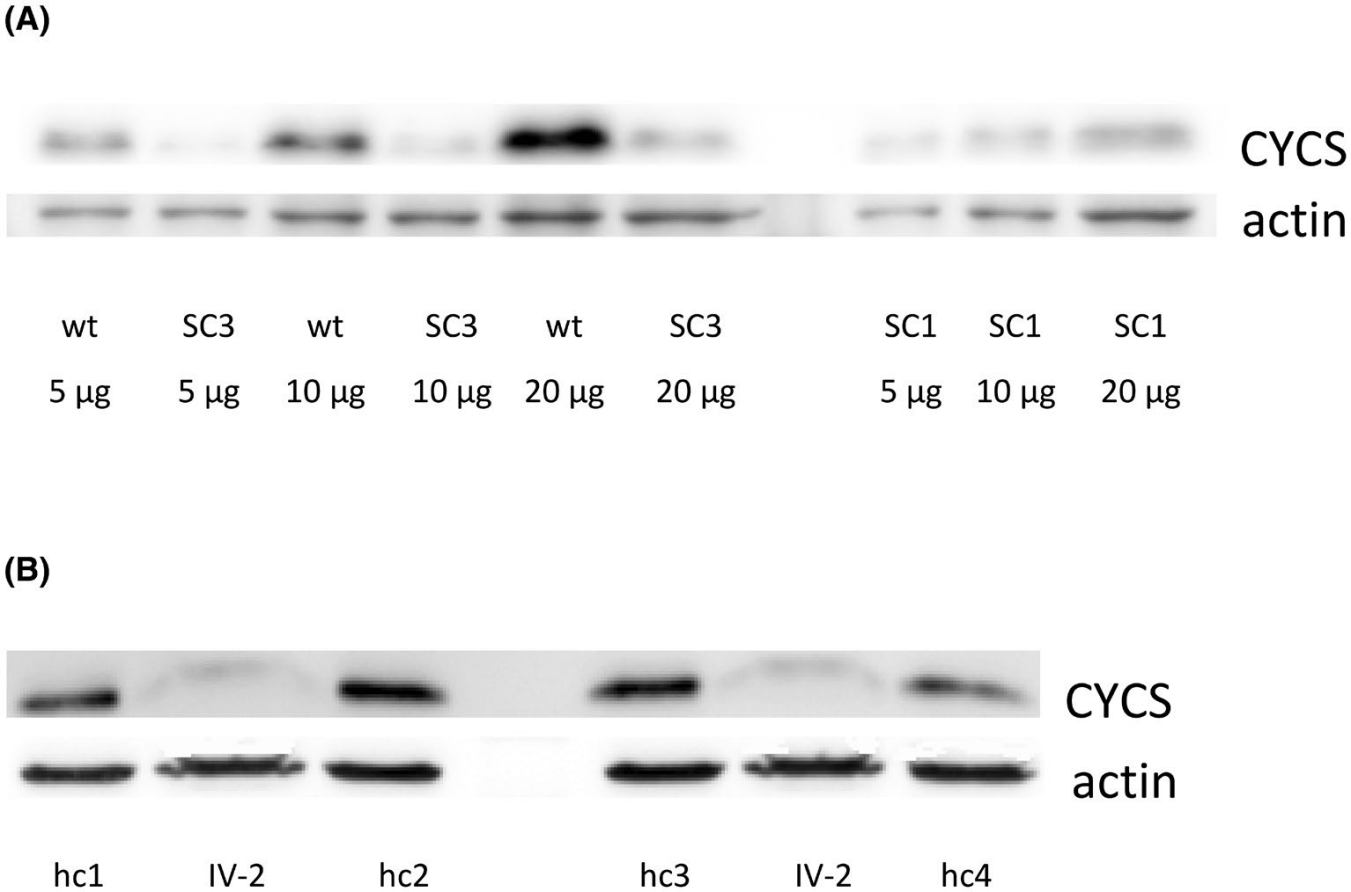
Expression of CYCS was decreased in: (A) SC1 and SC3 MEG‐01 p.(Thr20Ile) knock‐in clones compared to wt (the amount of protein used is indicated under electrophoretogram) and (B) in platelets of thrombocytopenic patient IV‐2 compared to four different healthy controls (hc1–hc4).

To analyse the functional impact of the variant p.(Thr20Ile), we used the CRISPR/Cas9 technology and introduced this variant into the *CYCS* gene in the MEG‐01 cell line. CYCS expression was reduced to 37% (*p* < 0.002) and 48% (*p* < 0.003) in two p.(Thr20Ile) knock‐in MEG‐01 clones SC1 and SC3, respectively, compared to wt (Figure 3A) and was also reduced to 43% (*p* < 0.002) in platelets from thrombocytopenic patient IV‐2 compared to healthy controls (Figure 3B). A paired *t*‐test was used.

### The variant p.(Thr20Ile) and markers of differentiation

The effects of the variant p.(Thr20Ile) on markers of blood cell differentiation were studied in MEG‐01 p.(Thr20Ile) knock‐in clones. The variant did not affect: (1) adhesion, shape and size assessed by regular light microscope inspection; (2) ploidy and CD41 and CD61 cell surface antigen expression assessed by flow cytometry (Figures S2 and S3). Out of the analysed surface markers, only CD9 expression was increased by 40% in SC1 (*p* = 0.003) and by 31% in SC3 (*p* = 0.022) MEG‐01 p.(Thr20Ile) knock‐in clones compared to wt (Figure S3). The platelet immunophenotype of patient IV‐2 was normal (data not shown).

### The variant p.(Thr20Ile) increases mitochondrial activity

**FIGURE 4:**
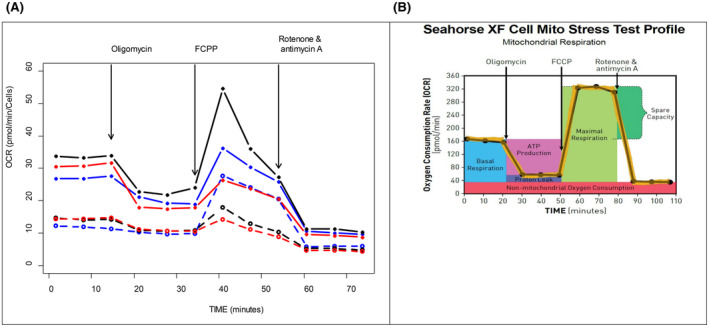
(A) Basal and maximal respiration were increased in MEG‐01 p.(Thr20Ile) knock‐in clone (solid lines) compared to wt (dashed lines). The results of three independent experiments are shown, each in a different colour. The points on the graph represent the median of the three values. (B) The Seahorse XF Cell Mito Stress Profile. FCCP, carbonyl cyanide‐4‐(trifluoromethoxy)phenylhydrazone.

Cytochrome c is an essential component of the respiratory electron transport chain in mitochondria. Therefore, the Seahorse XFp analyser was used to investigate how mitochondrial activity is affected by the presence of the variant p.(Thr20Ile). The MEG‐01 p.(Thr20Ile) knock‐in clone showed increased basal respiration by 164% (*p* < 5.9 × 10^−7^) and maximal respiration by 111% (*p* = 0.015) compared to wt (Figure 4; Figure S4), indicating that not only was basal respiration lower in the wt cells compared with the mutants but they also had lower spare respiratory capacity. Generally, they exhibited a very low amplitude of changes upon additions of oligomycin and FCCP.

### The variant p.(Thr20Ile) interferes with caspase activation

**FIGURE 5:**
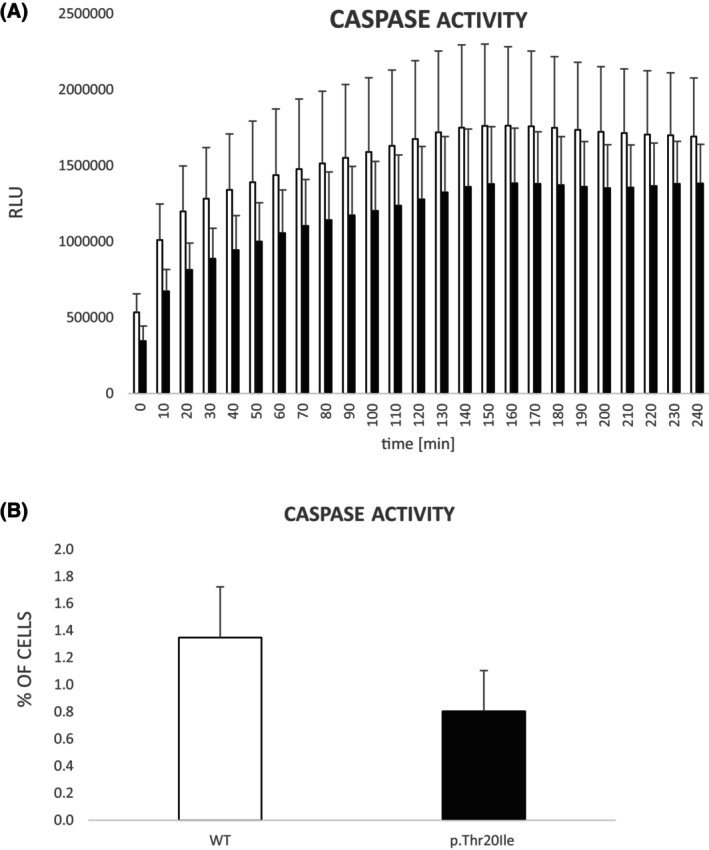
Caspase activity was decreased in MEG‐01 p.(Thr20Ile) knock‐in clone (filled bars) compared to wt (open bars) in (A) cytoplasmic lysates and (B) intact cells assessed by flow cytometry. Mean ± SD values are shown. RLU, relative luminescence unit.

The effect of the variant p.(Thr20Ile) on caspase activity was studied in the MEG‐01 p.(Thr20Ile) knock‐in clone by two methods. The variant reduces caspase activity to 75% in cytoplasmic lysates (*p* < 0.049; cell‐free assay; Figure 5A) and in the intact MEG‐01 cells to 60% (*p* < 0.012; flow cytometry; Figure 5B) compared to wt. In the case of the cytoplasmic lysates, post hoc tests proved statistically significant differences in early time points (0–40 min), followed by trends in time points 50–70 min. In the case of intact cells, a paired *t*‐test was used.

## DISCUSSION

This study identified a novel *CYCS* variant, p.(Thr20Ile), in a family with autosomal‐dominant non‐syndromic thrombocytopenia. This variant segregates with thrombocytopenia across three generations, affects an evolutionary conserved amino acid position and is classified as likely pathogenic according to the ACMG/AMP criteria. Functional analyses show that the variant decreases CYCS expression and caspase activation while increasing mitochondrial respiration.

Platelets from affected individuals display normal surface antigen expression, morphology and size, consistent with the THC4 phenotype. The seven *CYCS* gene variants described^1^, ^2^, ^3^, ^4^, ^5^, ^6^, ^7^ have been associated with mild to moderate thrombocytopenia. Interestingly, in the family analysed here, the severity of thrombocytopenia worsens across generations (Table 1). The cause of this is unknown, as we found no association between immunological problems, other variants identified by WES and thrombocytopenia.

Our in silico crystal structure analysis reveals that the p.(Thr20Ile) substitution disrupts the highly conserved contact with Lys26, potentially destabilizing the local structure (Figure 2). This destabilization may lead to reduced protein levels, as observed in both the MEG‐01 model and the patient's platelets (Figure 3). Similarly, the p.(Lys101del) variant also shows reduced CYCS expression,^7^ which can negatively impact megakaryocyte maturation.^19^ However, in our model, megakaryocytic differentiation was largely unaffected by this variant, with only an increase in CD9 expression (Figure S3). Notably, the p.(Thr20Ile) variant is uniquely located in the first Ω‐loop (residues 14–36) (Figure 1B), which may be crucial for protein function and stability^20^ and the severity of thrombocytopenia.^8^

To further investigate the origin of thrombocytopenia, we focused on CYCS's roles in the respiratory chain and apoptosis. MEG‐01 p.(Thr20Ile) knock‐in cells exhibited decreased caspase activity both in cell‐free assays and by flow cytometry (Figure 5) and increased mitochondrial respiration (Figure 4). This increase, along with a decrease in CYCS expression, suggests that CYCS levels may not correlate with mitochondrial oxygen consumption. The reduction in caspase activity is pronounced (up to 75% and 60% depending on the method) and could therefore contribute to the mild clinical manifestations of THC4.

The variant contrasts with others, such as: (1) p.(Gly42Ser), which did not influence mitochondrial respiration and increased caspase activity in U937 cell cytosol after the external addition of cytochrome c^2^; (2) p.(Tyr49His), which reduced mitochondrial respiration and likely enhanced caspase activity, as it increases the apoptotic response to staurosporine in mouse fibroblasts^3^; and (3) p.(Lys101del), which also reduced mitochondrial activity in a yeast model system.^7^

There is substantial evidence that humans' megakaryocyte differentiation and platelet formation depend on caspase activity. Platelet production coincides with apoptosis in mature primary human megakaryocytes.^21^ Studies have shown that localized caspase‐3 and caspase‐9 activation is crucial for proplatelet formation in CD34^+^‐derived megakaryocytes, as demonstrated by using caspase‐specific inhibitors.^10^ Similarly, in the MEG‐01 cell line, caspase activation also promotes platelet production.^11^

Our findings reveals that variant p.(Thr20Ile) segregates with thrombocytopenia in the described family, reducing CYCS expression and interfering with caspase activation. This conclusion supports the hypothesis that CYCS and caspases are involved in human thrombopoiesis. Variant p.(Thr20Ile) is the first thrombocytopenia‐causing variant in the human *CYCS* gene that is shown to downregulate caspase activity.

In conclusion, the novel germline thrombocytopenia‐causing CYCS variant p.(Thr20Ile) interferes with protein expression, increases mitochondrial activity and reduces basal caspase activity, a reduction that is distinct among thrombocytopenia‐causing *CYCS* gene variants. These findings provide new and unique insights into the mechanisms of human thrombopoiesis.

## AUTHOR CONTRIBUTIONS

JŠ, KSK, MŠ and SU were involved in conceptualization; MD and ŠP were involved in resources; MD and ŠP were involved in supervision; JŠ, MP, MS, LD, TL, VP, KR, ZV and IB were involved in investigation; LR, JŠ, KSK, MP and ZV were involved in formal analysis; MD, JT and JB were involved in variant interpretation and clinical diagnostics; JŠ was involved in writing original draft; KSK and MP were involved in writing review and editing. All authors have read and agreed to the published version of the manuscript.

## FUNDING INFORMATION

This work was supported by National Institute for Cancer Research (Program EXCELES) (LX22NPO5102)—funded by the European Union‐Next Generation EU; the Ministry of Health of the Czech Republic: NU20‐08‐00137 and conceptual development of research organization (FNBr, 65269705); Masaryk University (MUNI/A/1224/2022; MUNI/11/SUP/22/2020); European Regional Development Fund (Project ‘A‐C‐G‐T’) (CZ.02.1.01/0.0/0.0/16_026/0008448).

## CONFLICT OF INTEREST STATEMENT

No conflicts of interest to disclose.

## ETHICS APPROVAL STATEMENT

Patients or their legal guardians signed written informed consent in accordance with the Declaration of Helsinki and the research protocol was approved by institutional ethics committees of: Masaryk University (#EKV‐2019‐057, approved on 26 June 2019) and the University Hospital Brno (#22‐120 619/EK, approved on 12 June 2019).

## Supporting information


Figure S1.
Figure S2.
Figure S3.
Figure S4.
Table S1.
Table S2.

## Data Availability

The data used to support the findings of this study are available from the corresponding author upon request (for original data, contact jiri.stika@ceitec.muni.cz).
