# Supplementary material for: A novel thrombocytopenia‐4‐causing CYCS gene variant decreases caspase activity: Three‐generation study
Source: Br J Haematol. 2024 Aug 27;205(6):2450–8. doi: 10.1111/bjh.19694 (PMC11637729; doi:10.1111/bjh.19694)
Supplement: Supplementary file 1 — Figure S1. Figure S2. Figure S3. Figure S4. Table S1. Table S2. [file BJH-205-2450-s001.docx]

**SUPPLEMENTARY DATA**

**Figure S1** Platelet morphology


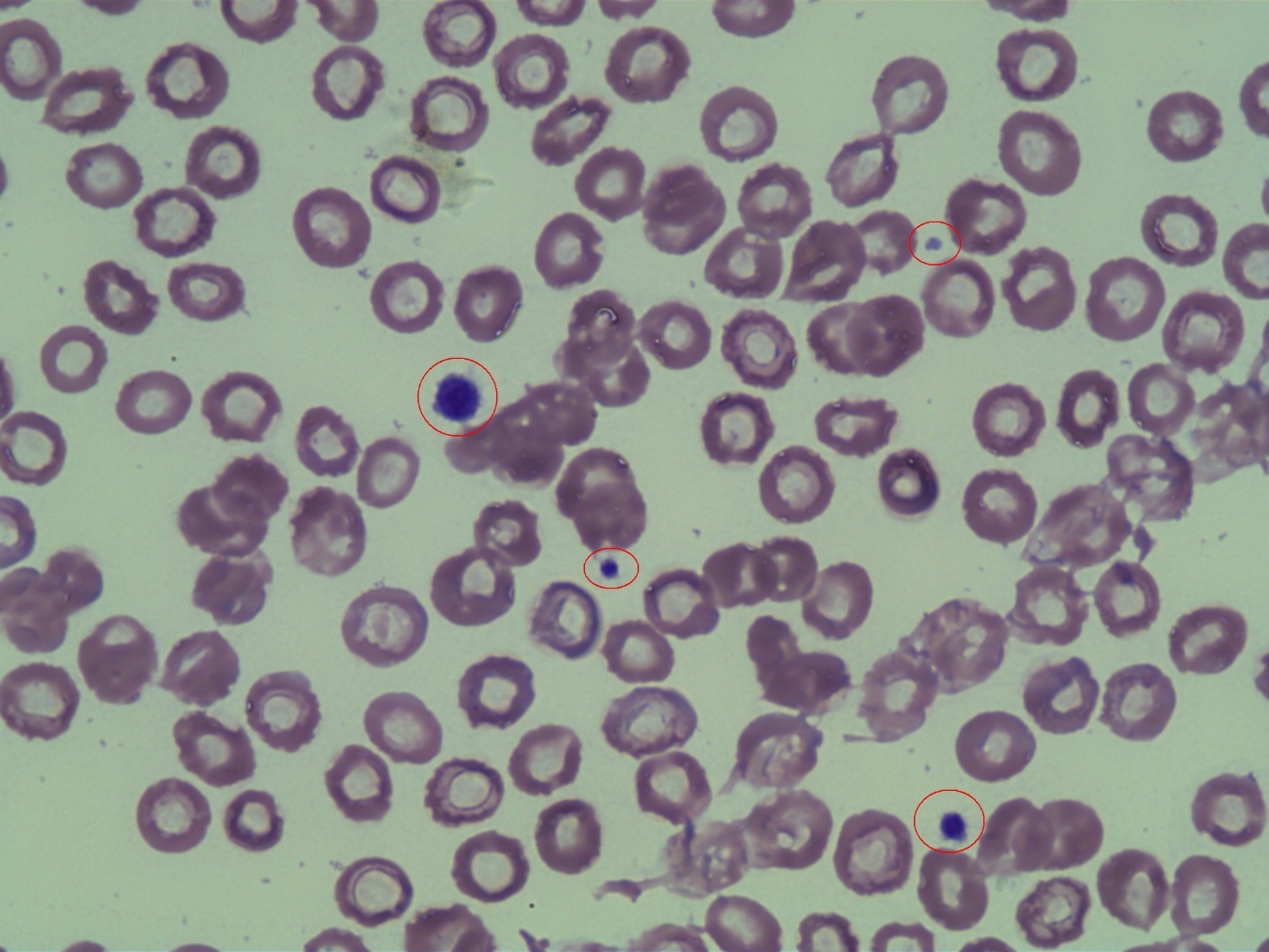


Figure S1: Morphology of thrombocytopenic patients’ platelets was normal. Representative image of peripheral blood smear from individual IV-2 is shown. Platelets are marked by red ellipses.

**Table S1** Virtual panel of thrombocytopenia-related and hematopoiesis-related genes

| ABCA12 | GATA2 | P2RY1 | SLC9A3R1 | RPL23 | DNASE1 | MMAB | SALL4 |
| --- | --- | --- | --- | --- | --- | --- | --- |
| ABCB4 | GDI2 | P2RY13 | SLC9A3R2 | RPL36 | DOCK2 | MMACHC | SAMHD1 |
| ABCC4 | GFI1 | PADI2 | SLC25A38 | RPS15 | DOCK6 | MMUT | SARS2 |
| ABCG5 | GFI1B | PDE2A | SLFN14 | RPS27A | DZIP1L | MOGS | SASH3 |
| ABCG8 | GNA12 | PDE3A | SMAD1 | SLC25A38 | EFL1 | MPIG6B | SAT1 |
| ACSL4 | GNA13 | PDE4D | SMAD6 | SRP72 | ELF4 | MRAS | SC5D |
| ACTN1 | GNAI1 | PDE5A | SNAP23 | STN1 | ENPP1 | MS4A1 | SCARB2 |
| ACVRL1 | GNAI2 | PDPK1 | SNAP25 | F2 | EOGT | MTHFD1 | SCN10A |
| ADCY3 | GNAQ | PDZD3 | SNAP29 | F5 | EPB42 | MTOR | SCN11A |
| ADCY6 | GNAZ | PDZK1 | SNAPIN | MTHFR | EPHB2 | MVK | SCN9A |
| ADCY7 | GNB2 | PEAR1 | SNX1 | PROC | ERBB3 | MYORG | SEC24C |
| ADORA2B | GNB3 | PECAM1 | SRA1 | PROS1 | ERCC6L2 | MYSM1 | SF3B1 |
| ADRA2A | GNE | PGM3 | SRC | F8 | ESCO2 | NAA10 | SH2D1A |
| ADRA2B | GNG11 | PHOX2A | SRF | F9 | FARS2 | NABP1 | SKIC2 |
| ADRBK1 | GNG12 | PIK3CA | STIM1 | F11 | FAS | NBN | SKIC3 |
| AK3 | GNG13 | PIK3CB | STOM | ABCA1 | FASLG | NFKB1 | SLC19A2 |
| AKT1 | GNG5 | PIK3R1 | STX12 | ABCC6 | FCGR2B | NFKB2 | SLC20A2 |
| AKT2 | GP5 | PIK3R3 | STX2 | ABCD4 | FCGR2C | NHEJ1 | SLC35A1 |
| ALOX12 | GP9 | PIK3R5 | STX4 | ABL1 | FCGR3B | NIPBL | SLC37A4 |
| ANKRD12 | GP1BA | PLA2G4C | STX6 | ACAD9 | FIP1L1 | NLRC4 | SLC39A7 |
| ANKRD18A | GP1BB | PLCB2 | STX7 | ACAT1 | FLT1 | NLRP3 | SLC46A1 |
| ANKRD18B | GRAP2 | PLCB3 | STXBP1 | ACP5 | FOCAD | NOS3 | SLC7A7 |
| ANKRD26 | GRB2 | PLCG2 | STXBP3 | ADA | FOXP3 | NPM1 | SLF2 |
| ANKRD33 | GRK5 | PLDN | STXBP4 | ADA2 | FYB1 | NRAS | SMARCAL1 |
| AP3D1 | GRK6 | PPP1CA | STXBP5L | ADAR | GALC | NSUN2 | SMARCD2 |
| AP3M1 | GUCY1A3 | PPP1CB | STXBP6 | ADH5 | GBA1 | NUMA1 | SMC5 |
| AP3S1 | GUCY1B3 | PPP1CC | SUZ12 | AFG2A | GIMAP5 | OCLN | SMPD1 |
| APC | HOOK3 | PPP1R12A | SYK | AGK | GNA14 | OCRL | SNX10 |
| ARHGAP1 | HOXA11 | PPP1R12C | SYTL3 | ALG12 | GNAS | OSTM1 | SOCS1 |
| ARHGAP17 | HPS1 | PPP1R14A | SYTL4 | ALG8 | GUCY1A1 | OTUD5 | SOS1 |
| ARHGAP32 | HTR2A | PPP1R2 | TAL1 | AMN | HEATR3 | P4HA2 | SOS2 |
| ARHGAP6 | CHD3 | PRKACA | TAOK1 | ANKRD11 | HELLPAR | PCCA | SP110 |
| ARHGDIA | INPP5D | PRKACB | TEC | APOE | HIRA | PCCB | SPP1 |
| ARHGDIB | ITGA5 | PRKACG | TGFBR3 | ARHGAP31 | HLA-B | PDCD1 | SPRED2 |
| ARHGEF12 | ITGA2B | PRKARA2 | THPO | ARHGEF1 | HLA-DQA1 | PDGFB | SRP54 |
| ARHGEF3 | ITGB1 | PRKAR2A | TLN1 | ARPC1B | HLA-DQB1 | PDGFRB | SRSF2 |
| ARRB1 | ITGB3 | PRKCA | TLR2 | ARVCF | HLA-DRB1 | PEPD | STAT1 |
| ASPN | ITPR1 | PRKCB | TMCC2 | ASAH1 | HLCS | PHGDH | STAT2 |
| BAK1 | KIAA1109 | PRKCD | TPM1 | ASXL1 | HMGCL | PIGA | STAT3 |
| BCL2L1 | KIAA2018 | PRKCQ | TPM4 | ATP6AP1 | HMOX1 | PKHD1 | STAT4 |
| BET1L | LAIR1 | PRKD1 | TRAF4 | ATP6V1B2 | HPS3 | PLAU | STAT5B |
| BLOC1S1 | LPAR1 | PRKG1 | TREML1 | ATP7B | HPS5 | PLEKHM1 | STING1 |
| BLOC1S2 | LRG | PRKG2 | TRPM7 | ATRX | HPS6 | PLOD3 | STOX1 |
| BLOC1S4 | LTBP1 | PTEN | TTC37 | BANK1 | HSCB | PML | STT3B |
| BMP4 | LY6G6F | PTGIR | TTF2 | BCR | ICOS | PMM2 | TALDO1 |
| BTBD9 | LYN | PTGS1 | TUBA3C | BLK | IFIH1 | PNP | TBC1D24 |
| C14orf133 | MAP2K2 | PTK2 | TUBB1 | BRAF | IFNG | POLRMT | TBL1XR1 |
| C19orf55 | MAP2K4 | PTPN1 | UNC13A | BTNL2 | IFNGR1 | POMP | TBX1 |
| C20orf42 | MAP3K9 | PTPN12 | UNC13B | C3 | IGHG1 | PPIL1 | TBXAS1 |
| C6orf25 | MAPK1 | PTPN18 | VAMP2 | C4A | IKZF5 | PRDX1 | TCIRG1 |
| CD226 | MAPK13 | PTPN2 | VAMP3 | C4B | IL10 | PRF1 | TCN2 |
| CLEC1B | MAPK14 | PTPN6 | VAMP7 | CA2 | IL6ST | PRIM1 | TET2 |
| CLEC4F | MAPK8 | PTPN7 | VAMP8 | CALR | IL7R | PSAP | TFRC |
| CNO | MDS1 | PTPN9 | VAV1 | CASP10 | IRAK1 | PSMB4 | TGFB1 |
| CSK | MECOM | PTPRA | VAV2 | CD109 | IRF2BP2 | PSMB8 | THBD |
| CTTN | MKL1 | PTPRC | VAV3 | CD19 | IRF5 | PSMB9 | TLR7 |
| CYCS | MLH1 | PTPRJ | VPS8 | CD40LG | IRF8 | PSTPIP1 | TLR8 |
| DAAM1 | MLK1 | RAB27A | VPS11 | CD46 | ITGAM | PTPN22 | TMEM165 |
| DDX41 | MLPH | RAB27B | VPS16 | CD55 | ITK | PXK | TNFAIP3 |
| DIAPH1 | MMP17 | RAB38 | VPS18 | CD81 | IVD | RAG1 | TNFRSF13B |
| DIAPH2 | MNX1 | RAB4A | VPS39 | CDC40 | JAM2 | RAG2 | TNFRSF13C |
| DIAPH3 | MPL | RABGGTA | VPS41 | CDC42 | JAZF1 | RARA | TNFSF11 |
| DNAH11 | MRPS34 | RAC1 | VPS4B | CFB | KARS1 | RASA2 | TNFSF12 |
| DNM1L | MUC16 | RAF1 | VPS52 | CFH | KCNJ1 | RASGRP1 | TNFSF4 |
| DNM2 | MUC2 | RAI1 | VPS8 | CFHR1 | KCNN4 | RBPJ | TNIP1 |
| DNM3 | MUTED | RAP1B | VWF | CFHR3 | KDM6A | RECQL | TOM1 |
| EFNB1 | MYB | RAP1GAP | WDR66 | CFI | KIAA0319L | REL | TPP2 |
| EPHA4 | MYH9 | RAP1GAP2 | ZFPMI | CIITA | KIF15 | RFX5 | TREX1 |
| EPHB1 | MYH10 | RAP1GDS1 | ATG2B | CISD2 | KIT | RFXANK | TRNT1 |
| ERG | MYH13 | RBM8A | ATM | CLCN7 | KMT2D | RFXAP | TTC7A |
| ETV6 | MYL9 | RGS10 | BLM | CLPB | KRAS | RIT1 | TUBA8 |
| EXOC1 | MYLK | RGS18 | CDAN1 | COG1 | LACC1 | RNASEH2A | TYMS |
| F2R | MYLK2 | RGS19 | CEBPA | COG4 | LARS2 | RNASEH2B | UBA1 |
| F2RL3 | MYO18B | RGS20 | DDX41 | COG6 | LBR | RNASEH2C | UBE2L3 |
| FANCD2 | MYO3A | RGS9 | EPCAM | COL4A5 | LCK | RNU7-1 | UFD1 |
| FARP2 | MYO5A | RHOA | GSKIP | COMT | LIPA | RPL18 | UNC13D |
| FCER1G | MYO5B | RHOC | HAX1 | CORIN | LMBRD1 | RPL35 | UQCRFS1 |
| FERMT1 | NAPA | RHOF | CHEK2 | CR2 | LRBA | RPL8 | UROS |
| FGD3 | NAPG | ROCK1 | MLH1 | CTLA4 | LSM11 | RPL9 | USB1 |
| FGR | NBEA | ROCK2 | MSH2 | CTNNBL1 | LZTR1 | RPS14 | USP18 |
| FHOD1 | NBEAL2 | RPL15 | MSH6 | CUBN | MADD | RPS15A | VPS45 |
| FLI1 | NFE2 | SCAMP2 | NBS1 | DCLRE1C | MAGT1 | RPS20 | WARS2 |
| FLII | NIPSNAP3A | SCAMP5 | NF1 | DEF6 | MAP2K1 | RPSA | WDR1 |
| FMNL1 | NOX1 | SCFD1 | PAX5 | DGKE | MARS1 | RRAS | WFS1 |
| FMNL3 | NRG3 | SELP | PMS2 | DGUOK | MECP2 | RRAS2 | WIPF1 |
| FRMPD1 | NSF | SERPINE2 | RBBP6 | DHFR | MED12 | RREB1 | XIAP |
| FYB | NXF1 | SIRPA | RECQL2 | DLL4 | MGAT2 | RUNX1 | XPR1 |
| FYN | P2RX1 | SLC35D3 | RECQL4 | DNAJC21 | MMAA | RYR1 | ZAP70 |
|  |  |  |  |  |  | ZBTB16 | ZNFX1 |

**Table S2** Virtual panel of immunity-related genes

| *ABCB1* | *C9* | *CSF2RA* | *FOXN1* | *IL18BP* | *MAGT1* | *OSTM1* | *RELA* | *SPINK5* | *TNFRSF11A* |
| --- | --- | --- | --- | --- | --- | --- | --- | --- | --- |
| *ACD* | *CARD11* | *CSF2RB* | *FOXP3* | *IL1RN* | *MALT1* | *OTULIN* | *RELB* | *SPPL2A* | *TNFRSF13B* |
| *ACOD1* | *CARD14* | *CSF3R* | *FPR1* | *IL21* | *MAP3K14* | *PARN* | *RFX5* | *SRC* | *TNFRSF13C* |
| *ACP5* | *CARD16* | *CTC1* | *FYB1* | *IL21R* | *MAPK8* | *PEPD* | *RFXANK* | *SRP54* | *TNFRSF1A* |
| *ACTB* | *CARD9* | *CTLA4* | *G6PC3* | *IL22* | *MASP2* | *PGM3* | *RFXAP* | *SRP72* | *TNFRSF4* |
| *ADA* | *CARMIL2* | *CTPS1* | *G6PD* | *IL23R* | *MCM4* | *PIK3CD* | *RHOH* | *STAT1* | *TNFRSF9* |
| *ADA2* | *CASP10* | *CTSC* | *GATA2* | *IL2RA* | *MEFV* | *PIK3CG* | *RIPK1* | *STAT2* | *TNFSF11* |
| *ADAM17* | *CASP8* | *CXCR4* | *GFI1* | *IL2RB* | *MFAP5* | *PIK3R1* | *RMRP* | *STAT3* | *TNFSF12* |
| *ADAMTS3* | *CCBE1* | *CYBA* | *GIMAP6* | *IL2RG* | *MOGS* | *PLA2G4A* | *RNASEH2A* | *STAT5B* | *TNFSF13* |
| *ADAR* | *CD19* | *CYBB* | *GINS1* | *IL36RN* | *MRTFA* | *PLCG2* | *RNASEH2B* | *STIM1* | *TNFSF15* |
| *AICDA* | *CD247* | *CYBC1* | *GUCY2C* | *IL6* | *MS4A1* | *PLEKHM1* | *RNASEH2C* | *STK4* | *TOP2B* |
| *AIRE* | *CD27* | *CYCS* | *HAVCR2* | *IL6R* | *MSH6* | *PLG* | *RNF168* | *STN1* | *TP53* |
| *AK2* | *CD28* | *DBR1* | *HAX1* | *IL6ST* | *MSN* | *PMS2* | *RNF186* | *STX11* | *TPP1* |
| *ALPI* | *CD3D* | *DCLRE1B* | *HELLS* | *IL7* | *MTHFD1* | *PNP* | *RNF31* | *STXBP2* | *TPP2* |
| *ANGPT1* | *CD3E* | *DCLRE1C* | *HMOX1* | *IL7R* | *MVK* | *POLA1* | *RNU4ATAC* | *STXBP3* | *TRAC* |
| *ANKRD26* | *CD3G* | *DEF6* | *HPS1* | *INAVA* | *MYD88* | *POLD1* | *RORC* | *TAP1* | *TRAF3* |
| *ANKZF1* | *CD40* | *DKC1* | *HPS3* | *INO80* | *MYH9* | *POLD2* | *RPSA* | *TAP2* | *TRAF3IP2* |
| *AP1S1* | *CD40LG* | *DNAJC21* | *HPS4* | *IRAK1* | *MYOF* | *POLE* | *RTEL1* | *TAPBP* | *TREX1* |
| *AP1S3* | *CD46* | *DNASE1* | *HPS5* | *IRAK4* | *MYSM1* | *POLE2* | *SAMD9* | *TAZ* | *TRIM22* |
| *AP3B1* | *CD55* | *DNASE1L3* | *HPS6* | *IRF2BP2* | *NBAS* | *POLR3A* | *SAMD9L* | *TBK1* | *TRNT1* |
| *AP3D1* | *CD59* | *DNASE2* | *HS3ST6* | *IRF3* | *NBN* | *POLR3C* | *SAMHD1* | *TBX1* | *TTC37* |
| *APOL1* | *CD70* | *DNMT3B* | *HSPA1L* | *IRF4* | *NCF1* | *POLR3E* | *SART3* | *TCF3* | *TTC7A* |
| *ARHGEF1* | *CD79A* | *DOCK2* | *HYOU1* | *IRF5* | *NCF2* | *POLR3F* | *SBDS* | *TCIRG1* | *TYK2* |
| *ARPC1B* | *CD79B* | *DOCK8* | *CHD7* | *IRF7* | *NCF4* | *POMP* | *SEC61A1* | *TCN2* | *UNC119* |
| *ATG16L1* | *CD81* | *DPP9* | *ICOS* | *IRF8* | *NCKAP1L* | *PRDM1* | *SEMA3E* | *TERC* | *UNC13D* |
| *ATP6AP1* | *CD8A* | *DTNBP1* | *ICOSLG* | *IRF9* | *NCSTN* | *PRF1* | *SERPING1* | *TERT* | *UNC93B1* |
| *B2M* | *CDC42* | *DUOX2* | *IFIH1* | *IRGM* | *NFAT5* | *PRKCD* | *SH2D1A* | *TFRC* | *UNG* |
| *BACH2* | *CDCA7* | *EFL1* | *IFNAR1* | *ISG15* | *NFE2L2* | *PRKDC* | *SH3BP2* | *TGFB1* | *USB1* |
| *BCL10* | *CEBPE* | *EGFR* | *IFNAR2* | *ITGAM* | *NFKB1* | *PSEN1* | *SH3KBP1* | *TGFBR1* | *USP18* |
| *BCL11B* | *CFB* | *ELANE* | *IFNG* | *ITGB2* | *NFKB2* | *PSENEN* | *SHARPIN* | *TGFBR2* | *VPS13B* |
| *BLM* | *CFD* | *EPG5* | *IFNGR1* | *ITCH* | *NFKBIA* | *PSMA3* | *SKIV2L* | *THBD* | *VPS45* |
| *BLNK* | *CFH* | *ERBIN* | *IFNGR2* | *ITK* | *NFKBIB* | *PSMB10* | *SLC29A3* | *TICAM1* | *WAS* |
| *BLOC1S6* | *CFHR1* | *ERCC6L2* | *IGHM* | *JAGN1* | *NHEJ1* | *PSMB4* | *SLC35C1* | *TINF2* | *WASF2* |
| *BTK* | *CFHR2* | *ETV6* | *IGKC* | *JAK1* | *NHP2* | *PSMB8* | *SLC37A4* | *TIRAP* | *WDR1* |
| *C1QA* | *CFHR3* | *EXTL3* | *IGLL1* | *JAK3* | *NLRC4* | *PSMB9* | *SLC39A7* | *TLR1* | *WIPF1* |
| *C1QB* | *CFHR4* | *F12* | *IKBKB* | *KDM6A* | *NLRP1* | *PSMG2* | *SLC46A1* | *TLR10* | *WRAP53* |
| *C1QC* | *CFHR5* | *FAAP24* | *IKBKG* | *KMT2A* | *NLRP12* | *PSTPIP1* | *SLC6A20* | *TLR2* | *XIAP* |
| *C1R* | *CFI* | *FADD* | *IKZF1* | *KMT2D* | *NLRP3* | *PTCRA* | *SLC7A7* | *TLR3* | *ZAP70* |
| *C1S* | *CFP* | *FAS* | *IL10* | *KNG1* | *NOD2* | *PTEN* | *SLC9A3* | *TLR4* | *ZBTB24* |
| *C2* | *CIB1* | *FASLG* | *IL10RA* | *LACC1* | *NOP10* | *PTPRC* | *SLCO2A1* | *TLR5* | *ZNF341* |
| *C3* | *CIITA* | *FAT4* | *IL10RB* | *LAMTOR2* | *NOS2* | *RAB27A* | *SMARCAL1* | *TLR6* | *ZNFX1* |
| *C5* | *CLCN7* | *FCGR3A* | *IL12B* | *LAT* | *NOX1* | *RAC2* | *SMARCD2* | *TLR7* |  |
| *C6* | *CLPB* | *FCGR3B* | *IL12RB1* | *LCK* | *NPC1* | *RAG1* | *SMURF1* | *TLR8* |  |
| *C7* | *COG6* | *FCN3* | *IL12RB2* | *LIG1* | *NSMCE3* | *RAG2* | *SNM1* | *TLR9* |  |
| *C8A* | *COL7A1* | *FERMT1* | *IL17A* | *LIG4* | *OAS1* | *RANBP2* | *SNORA31* | *TMC6* |  |
| *C8B* | *COPA* | *FERMT3* | *IL17F* | *LPIN2* | *OAS2* | *RASGRP1* | *SNX10* | *TMC8* |  |
| *C8G* | *CORO1A* | *FCHO1* | *IL17RA* | *LRBA* | *OAS3* | *RBCK1* | *SOCS4* | *TMEM173* |  |
| *C8orf34* | *CR2* | *FNIP1* | *IL17RC* | *LYST* | *ORAI1* | *REL* | *SP110* | *TNFAIP3* |  |

**Figure S2** Cell cycle analysis

Figure S2: Ploidy did not change in MEG-01 p.(Thr20Ile) knock-in clones (SC1 and SC3) compared to wt.

**Figure S3** Cell surface antigen expression in MEG-01

Figure S3: CD9 cell surface antigen expression was increased in SC1 and in SC3 MEG-01 p.(Thr20Ile) knock-in clones compared to wt. On the contrary, CD41 and CD61 cell surface antigen expression was not influenced by the presence of the variant p.(Thr20Ile). The one-way ANOVA with blocking variable (“experiment ID”) was applied followed by the Tukey HSD post hoc test. Mean + S.D. values are shown. Median fluorescence intensity (MFI).

**Figure S4** Mitochondrial activity - separate graphs for independent experiments


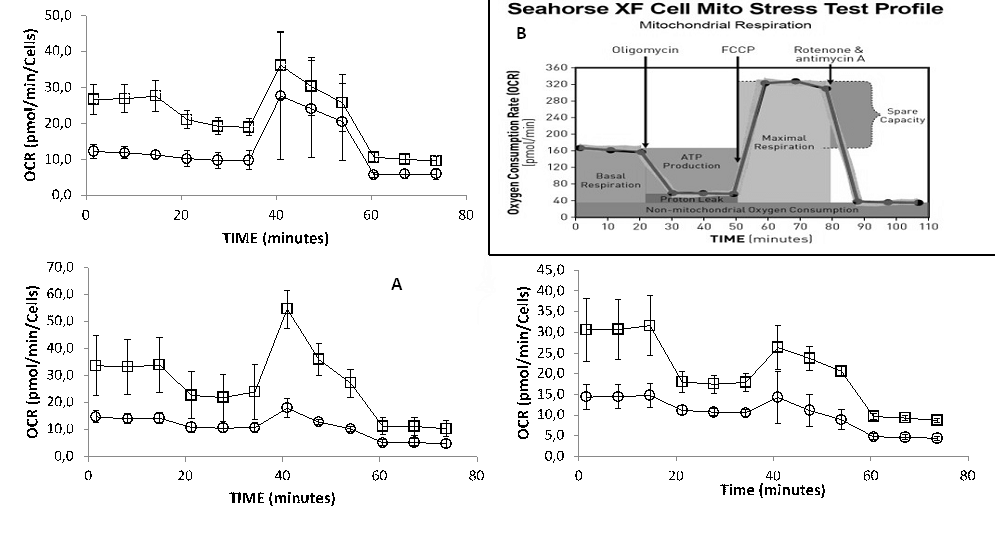


Figure S4: **A** Basal and maximal respiration were increased in MEG-01 p.(Thr20Ile) knock-in clone (squares) compared to wt (circles). Mean ± S.D. values are shown. **B** The Seahorse XF Cell Mito Stress Test Profile. Carbonyl cyanide-4-(trifluoromethoxy)phenylhydrazone (FCCP).
